# Baboon phylogeny as inferred from complete mitochondrial genomes

**DOI:** 10.1002/ajpa.22185

**Published:** 2013-01

**Authors:** Dietmar Zinner, Jenny Wertheimer, Rasmus Liedigk, Linn F Groeneveld, Christian Roos

**Affiliations:** 1 Cognitive Ethology Laboratory, German Primate Center37077 Göttingen, Germany; 2 Primate Genetics Laboratory, German Primate Center37077 Göttingen, Germany; 3 Courant Research Centre Geobiology, Georg-August-Universität Göttingen37077 Göttingen, Germany; 4 Gene Bank of Primates, German Primate Center37077 Göttingen, Germany

**Keywords:** *Papio*, evolution, Africa

## Abstract

Baboons (genus *Papio*) are an interesting phylogeographical primate model for the evolution of savanna species during the Pleistocene. Earlier studies, based on partial mitochondrial sequence information, revealed seven major haplogroups indicating multiple para- and polyphylies among the six baboon species. The most basal splits among baboon lineages remained unresolved and the credibility intervals for divergence time estimates were rather large. Assuming that genetic variation within the two studied mitochondrial loci so far was insufficient to infer the apparently rapid early radiation of baboons we used complete mitochondrial sequence information of ten specimens, representing all major baboon lineages, to reconstruct a baboon phylogeny and to re-estimate divergence times. Our data confirmed the earlier tree topology including the para- and polyphyletic relationships of most baboon species; divergence time estimates are slightly younger and credibility intervals narrowed substantially, thus making the estimates more precise. However, the most basal relationships could not be resolved and it remains open whether (1) the most southern population of baboons diverged first or (2) a major split occurred between southern and northern clades. Our study shows that complete mitochondrial genome sequences are more effective to reconstruct robust phylogenies and to narrow down estimated divergence time intervals than only short portions of the mitochondrial genome, although there are also limitations in resolving phylogenetic relationships. Am J Phys Anthropol, 2013. © 2012 Wiley Periodicals, Inc.

Baboons of the genus *Papio* have been regarded as a useful analog for hominin behavioral and biological evolution because their evolutionary history took place in parallel to hominins in similar African savanna habitats (Jolly,^1970^, ^2001^; Strum and Mitchell,^1987^; Elton,^2006^; Codron et al.,^2008^; Swedell and Plummer,^2012^). Extant baboons occur in large parts of sub-Saharan Africa outside the Central- and West African rainforests and are also found in Southwestern Arabia (Jolly,^1993^; Kingdon,^1997^; Groves,^2001^) (Fig. .1a). Representatives of the genus *Papio* are traditionally divided into five different species or morphotypes, based on morphological, ecological, and behavioral characteristics (Hill,^1970^). These are Guinea baboons (*P. papio*)*,* olive baboons (*P. anubis*)*,* hamadryas baboons (*P. hamadryas*)*,* yellow baboons (*P. cynocephalus*), and chacma baboons (*P. ursinus*). A similar taxonomic status as for these five forms is warranted for Kinda baboons (*P. kindae*) (Jolly,^1993^, ^2001^; Frost et al.,^2003^; Burell,^2008^; Zinner et al.,^2009^). Whether these types should be classified as subspecies of the superspecies *P. hamadryas* (Jolly,^1993^) or as distinct species (Groves,^2001^; Grubb et al.,^2003^) is still disputed, but recent studies recognize the six morphotypes as species (Zinner et al.,2011a, 2012).

**Fig 1:**
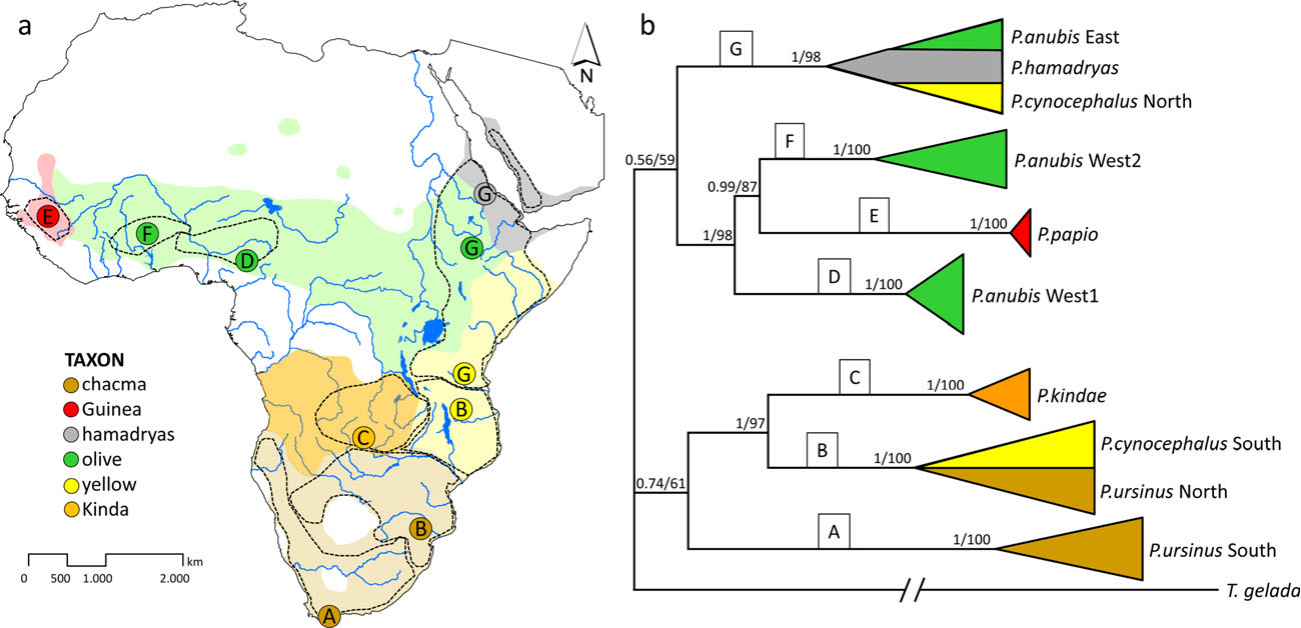
(a) Distribution of the six baboon species and the seven major mitochondrial haplogroups (A–G) (map based on Kingdon,^1997^; Jolly,^2007^; Zinner et al.,^2009^, in press), and (b) simplified phylogenetic relationships among the haplogroups (adapted from Zinner et al.,^2009^). In (a), dashed lines and colored circles indicate the distribution of the seven major mitochondrial haplogroups and the geographic provenance of samples used in this study (see also Table 1), respectively. In (b), numbers on branches represent support values from Bayesian inference and maximum-likelihood analysis, respectively.

Various studies tried to clarify the phylogenetic relationships within *Papio* by analyzing parts of the mitochondrial genome, such as the “Brown region” (Brown et al.,^1982^) or the cytochrome *b* gene (Newman etal.,^2004^; Wildman et al.,^2004^; Zinner et al.,^2009^; Keller et al.,^2010^). Zinner et al. (2009) attempted to resolve the phylogenetic relationships of baboons using both the “Brown region” and the cytochrome *b* gene, and revealed seven well-supported major haplogroups (Fig. .1b). The study revealed paraphylies in all species, except for Guinea and Kinda baboons, due to discordances between mitochondrial phylogeny and morphology and/or geographic distribution. However, a strong geographical signal with haplotypes of parapatric populations from different species clustering together was found (Zinner et al.,^2009^). Although the seven major haplogroups were strongly supported, phylogenetic relationships among them remained largely unresolved. Moreover, divergence time estimates indicated a fast radiation-like (star-like) splitting event into various baboon lineages, starting ∼ 2.09 million years ago (Ma) (Zinner et al.,^2009^), which might impede inferring the basal relationships with confidence.

It is plausible that genetic variation within the two studied mitochondrial loci was insufficient to infer the apparently rapid early radiation of baboons. The use of complete mitochondrial sequence information might allow a better resolution and stronger statistical support in the phylogenetic tree reconstruction and to narrow down the divergence time credibility intervals (DeFilippis and Moore,^2000^; Duchêne et al.,^2011^; Rokas and Carroll,^2005^). A similar approach was successfully applied in recent phylogenetic studies of other taxonomic groups, e.g., gibbons (Chan et al.,^2010^), colobines (Roos et al.,^2011^; Liedigk et al.,^2012^), squirrel monkeys (Chiou et al.,^2011^); woodpeckers (DeFilippis and Moore,^2000^), dolphins (Vilstrup et al.,^2011^), and bears (Yu et al.,^2007^). Hence, to test whether sequence information from the complete mitochondrial genome allows a better resolution of phylogenetic relationships and results in smaller divergence time credibility intervals than shorter mitochondrial fragments in baboons, we sequenced and analyzed complete mitochondrial genomes of ten baboons representing all six species and the seven major mitochondrial haplogroups, and compared them with published data.

## MATERIALS AND METHODS

### Sample collection

We used ten baboon fecal samples, which were collected at various sites in Africa (Fig. .1a and Table 1) for earlier phylogeographic studies (see Zinner et al.,^2009^ and Keller et al.,^2010^). We used two types of information to assign samples to respective species: (1) characteristic morphological cues and (2) biogeographic provenance of samples (see Zinner et al.,^2009^). While observing the animals directly in the field, we used pelage color, body size, general body form, carriage of the tail (curved or “broken”) for species identification (after Kingdon,^1997^). We further compared the appearance of the baboons in the field with pictures in Kingdon (1997). The ten baboon samples represent all six baboon species and the seven described haplogroups (Fig. .1b, Zinner et al.,^2009^): *P. ursinus* South (haplogroup A), *P. ursinus* North, and *P. cynocephalus* South (haplogroup B), *P. kindae* (haplogroup C), *P. anubis* West1 (haplogroup D), *P. papio* (haplogroup E), *P. anubis* West2 (haplogroup F), and *P. anubis* East, *P. hamadryas* and *P. cynocephalus* North (haplogroup G). The respective samples were randomly selected from collections of samples of the respective species and haplogroups used in an earlier analysis (Zinner et al.,^2009^). Fresh fecal material was preserved in 40 ml 75% ethanol and dry samples were stored directly on 40 ml silica gel in 50-ml tubes. We stored the samples at ambient temperature for up to 6 months before further processing. The geographic coordinates of the sampling locations were recorded with a GPS.

**Table 1:** Baboon samples

Species	Haplogroup	Site	Country	Long	Lat	Acc no
*P. anubis*	G	Managasha NP	Ethiopia	38.57125	8.96838	JX946196
*P. anubis*	D	Komoe NP	Côte d'Ivoire	−3.79000	8.80000	JX946198
*P. anubis*	F	Gashaka-Gumti NP	Nigeria	11.58333	7.31667	JX946197
*P. cynocephalus*	G	Mikumi NP	Tanzania	37.16463	−7.34651	JX946199
*P. cynocephalus*	B	Amani	Tanzania	37.51363	−11.26054	JX946200
*P. hamadryas*	G	Furrus	Eritrea	38.97115	15.01148	JX946201
*P. papio*	E	Niokolo Koba NP	Senegal	−12.76667	12.88333	JX946203
*P. kindae*	C	Kasanka NP	Zambia	30.25202	−12.59059	JX946202
*P. ursinus*	B	DeHoop NR	South Africa	20.40658	−34.45621	JX946204
*P. ursinus*	A	Blyde River	South Africa	30.79000	−24.68000	JX946205

NP: national park; NR: nature reserve.

Species, haplogroup affiliation, geographical location and GenBank accession numbers of studied baboons.

Our study complied with protocols approved by the respective authorities in countries of origin, and adhered to the legal requirements of the countries in which research was conducted. The study was carried out in compliance with respective animal care regulations and the principles of the American Society of Primatologists and the German Primate Center for the ethical treatment of nonhuman primates.

### Laboratory methods

DNA from fecal material was extracted using the QIAamp DNA Stool Mini Kit (Qiagen, Germany) following manufacturer's protocol with some modifications (Yang et al.,^2012^). Because of degradation of the DNA extracted from feces, mitochondrial genomes were amplified via 5–25 overlapping fragments, and nested PCRs with an average length of 1,000 bp were sequenced on an ABI 3130*xL* sequencer. Respective laboratory methods are outlined in detail in Roos et al. (2011) and Liedigk et al. (2012). To prevent crossindividual contamination, laboratory procedures followed described standards (Roos et al.,^2008^). Accordingly, DNA extraction, PCR, gel extraction, and sequencing were performed in separate laboratories and randomly repeated after several months, while always only one individual was tested. Sequences from independent analyses were identical. Because only fecal material was used, a contamination of the dataset with nuclear integrations of mitochondrial fragments (“numts”) can be regarded as minimal, because nuclear DNA is highly degraded in feces (Thalmann et al.,^2004^). In fact, test amplifications of the autosomal intron 3 of the serum albumin gene (ALB3) revealed positive PCR amplifications of only 200–400 bp from the 10 baboon DNAs. Further, no multiple amplifications of different copies were detected by direct sequencing of PCR products and overlapping fragments were identical as revealed by visually inspecting electropherograms of all sequences. Complete mitochondrial genome sequences were assembled with GeneiousPro 5.4 (Drummond et al.,^2011^) and annotated with the online program DOGMA (Wyman et al.,^2004^). According to DOGMA and manual verification, all protein-coding genes are correctly transcribed, and rRNAs and tRNAs are able to form their typical secondary structure. Sequences were deposited in GenBank (for accession numbers see Table 1).

### Statistical analysis

For phylogenetic analysis, additional orthologous sequences of other primate taxa deposited in GenBank were added. The final dataset comprised 18 sequences including ten baboons, four other cercopithecines (*Theropithecus gelada* [FJ785426], *Macaca sylvanus* [AJ309865], *M. mulatta* [JQ821843]*, Chlorocebus aethiops* [AY863426]), one colobine (*Colobus guereza* [AY863427]), and three hominoid species (*Homo sapiens* [X93334], *Pan troglodytes* [D38113], *Pongo abelii* [X97707]), that were used as outgroup taxa. Sequences were aligned with Muscle 3.7 (Edgar,^2004^) and manually corrected. For phylogenetic analyses, two different datasets were generated. The first dataset (mtDNA1) consists of the complete mitochondrial genome in which only poorly aligned positions and indels were removed with Gblocks 0.91b (Castresana,^2000^) using default settings. The second dataset (mtDNA2), generated in Mesquite 2.75 (Maddison and Maddison,^2011^), included only the 12 protein-coding genes on the heavy strand.

Phylogenetic tree reconstructions were conducted with maximum-likelihood (ML) and Bayesian algorithms, using respectively the programs GARLI 2.0 (Zwickl,^2006^) and MrBayes 3.1.2 (Huelsenbeck et al.,^2001^; Ronquist and Huelsenbeck,^2003^). For all reconstructions, the best-fit model of nucleotide substitution was chosen with the Bayesian information criterion (BIC) in jModeltest 2.1 (Posada,^2009^) (Supporting Information Table S1). For the mtDNA2 dataset, each locus was treated separately and each with its own substitution model. For ML reconstructions in GARLI, only the models were specified, while all other settings were left at their default value. Respective internal node support was assessed by bootstrap analyses with 500 replicates and majority-rule consensus trees were calculated in PAUP* 4.0b10 (Swofford,^2003^). For Bayesian analyses, we applied four independent Markov Chain Monte Carlo (MCMC) runs with the default temperature of 0.2. We ran four repetitions for 10 million generations with tree and parameter sampling every 100 generations. Acceptance rates were in the optimal range of 10–70%. The adequacy of a 25% burn-in and convergence of all parameters was checked via the uncorrected potential scale reduction factor (PSRF) (Gelman and Rubin,^1992^) as estimated by MrBayes and by inspecting the trace of the parameters across generations using the software TRACER 1.5 (Rambaut and Drummond,^2007^). Whether posterior split probabilities were also converging was examined with AWTY (Nylander et al.,^2008^). Posterior probabilities and a phylogram with mean branch lengths were calculated from the posterior density of trees. Alternative phylogenetic positions of the *P. ursinus* South haplogroup among baboons, and various alternative relationships among the *P. anubis* West1, *P. anubis* West2 and *P. papio* haplogroups were evaluated with the Kishino–Hasegawa (Kishino and Hasegawa,^1989^) and Shimodaira–Hasegawa (Shimodaira and Hasegawa,^1999^) tests with full optimization and 1,000 bootstrap replicates in PAUP*.

Divergence ages from both mitochondrial datasets were estimated in BEAST 1.6.1 (Drummond and Rambaut,^2007^) with a relaxed molecular clock approach (Drummond et al.,^2006^). Therefore, we assumed a relaxed lognormal model of lineage variation and a birth–death process prior for branching rates. The mtDNA2 dataset was partitioned treating each locus separately and each with its own substitution model, while dataset mtDNA1 was regarded as one partition. Five fossil-based calibration points were applied with a normal distribution prior for respective nodes: the split between *Homo* and *Pan* 6.5 Ma with a 95% credibility interval (CI) of 0.5 Ma (Vignaud et al.,^2002^; Brunet et al.,^2005^; Lebatard et al.,^2008^), the separation of *Pongo* from the *Homo* + *Pan* lineage 14 Ma and a 95% CI of 1.0 Ma (Kelley,^2002^), the split between *Theropithecus* and *Papio* 4 Ma (95% CI: 0.5 Ma) (Leakey,^1993^; Delson,^2000^), the split between *M. sylvanus* and *M. mulatta* 5.5 Ma (95% CI: 0.5 Ma) (Delson,^1980^), and the divergence of hominoids and cercopithecoids 26.5 Ma (95% CI: 2.5 Ma) (Zalmout et al.,^2010^; Pozzi et al.,^2011^). We ran four replicates for 25 million generations with tree and parameter sampling occurring every 1,000 generations. TRACER was used to assess the adequacy of a 10% burn-in and convergence of all parameters across generations. Subsequently, sampling distributions were combined (25% burn-in) with the software LogCombiner 1.6.1 and a consensus chronogram with node height distribution was generated and visualized with TreeAnnotator 1.6.1 and FigTree 1.3.1 (Rambaut,^2008^).

## RESULTS

Mitochondrial genome sequences were successfully amplified and sequenced from ten *Papio* individuals. All sequenced genomes consisted of 13 protein-coding genes, 2 rRNA genes, 22 tRNA genes, and the control region. The initial alignment comprising a total of 18 sequences had a length of 16,858 bp. After removing poorly aligned positions and indels, the mtDNA1 dataset had a length of 16,055 bp, while the mtDNA2 dataset, including only the 12 protein-coding genes on the heavy strand, was 10,854 bp in length.

**Figure 2:**
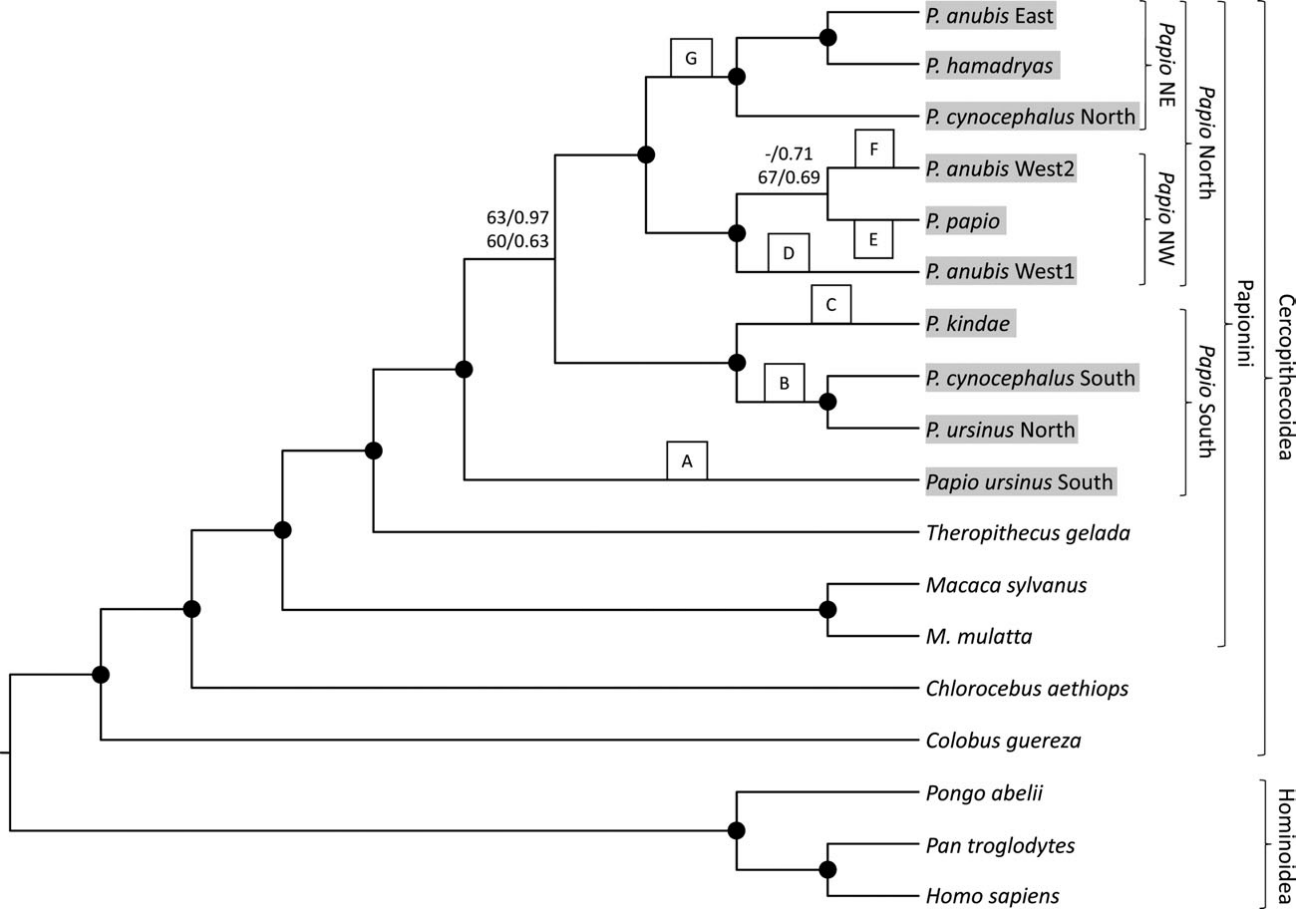
Phylogenetic relationships among baboons and outgroup taxa based on complete mitochondrial genome sequences. Black dots on nodes indicate ML bootstrap values of 99–100% and Bayesian posterior probabilities of 1.0; values below are given at respective branches (upper line: mtDNA1, lower line: mtDNA2). A–G indicates the seven major haplogroups in *Papio* according to Zinner et al. (2009).

Phylogenetic trees obtained from both datasets (mtDNA1 and mtDNA2) and derived from Bayesian inference and the ML algorithm yielded identical tree topologies and mainly well supported branching patterns (ML bootstrap values: 99–100%, Bayesian posterior probabilities: 1.0) (Fig. .2). The only exceptions are the phylogenetic position of *P. ursinus* South, and the relationships among the two western *P. anubis* and *P. papio*. According to our tree reconstructions, *P. ursinus* South diverged first and appears as sister lineage to all other baboons. However, statistical support is weak (ML bootstrap values: 60–63%, Bayesian posterior probabilities: 0.63–0.97) and alternative positions of *P. ursinus* South within the baboon clade (sister to either the southern or northern clades, or an unresolved trichotomy among these two clades and *P. ursinus* South) are statistically not rejected (*P* > 0.05). After this initial split, a major division occurred between the remaining southern and the northern lineages. Among the southern lineages, *P.kindae* split off before *P. ursinus* North and *P. cynocephalus* South separated. The northern lineages first divided into a northwestern and a northeastern clade. Within the latter, *P. cynocephalus* North diverged first from a clade consisting of *P. anubis* East and *P. hamadryas*. In the northwestern clade, *P. anubis* West1 is suggested to be the sister lineage to the *P. anubis* West2—*P. papio* clade. However, statistical support for this relationship is weak (ML bootstrap values: <50–67%, Bayesian posterior probabilities: 0.69–0.71) and alternative relationships (*P. anubis* West1 and West2 are sister taxa, *P. anubis* West1 is sister taxon to *P. papio*, or an unresolved trichotomy among the three lineages) are statistically not rejected (*P* > 0.05).

Divergence age estimates obtained from both datasets are highly similar albeit estimates from the mtDNA2 dataset are generally slightly younger than from the mtDNA1 dataset (Table 2). Accordingly, the divergence of *Papio* into the seven major haplogroups started 1.96–2.21 Ma and ended 0.68–0.74 Ma (for 95% credibility intervals see Table 2). The three lineages in haplogroup G diverged 0.31–0.39 and 0.16–0.23 Ma, respectively, and the two lineages in haplogroup B separated 0.68–0.74 Ma.

**Table 2:** Estimation of divergence ages in Ma (95% credibility intervals)

Split	mtDNA1 divergence ages	mtDNA2 divergence ages	Brown + cyt b divergence ages
Cercopithecoidea − Hominoidea	26.66 (24.29–28.95)	27.13 (24.75–29.57)	24.38 (18.98–30.33)
*Pongo − Homo + Pan*	13.61 (12.54–14.73)	13.74 (12.71–14.84)	13.74 (12.59–14.90)
*Homo – Pan*	6.18 (5.60–6.75)	6.19 (5.59–6.78)	6.43 (5.85–7.01)
*Colobus* – Cercopithecinae	17.94 (15.21–20.59)	19.05 (16.13–22.20)	15.63 (11.50–20.08)
*Chlorocebus* – Papionini	12.08 (10.38–13.82)	12.34 (10.54–14.11)	9.80 (7.72–12.07)
*Macaca − Papio + Theropithecus*	10.22 (8.73–11.73)	10.09 (8.62–11.55)	7.41 (6.42–8.46)
*Macaca sylvanus − M. mulatta*	5.54 (4.98–6.06)	5.51 (4.94–6.03)	4.75 (3.27–6.29)
*Papio – Theropithecus*	4.54 (4.04–5.04)	4.36 (3.87–4.86)	3.99 (2.92–5.09)
*P. ursinus* South − remaining baboons	2.21 (1.91–2.53)	1.96 (1.68–2.28)	
*Papio* southern clades – *Papio* northern clades			2.09 (1.54–2.71)
*P. ursinus* North + *P. cynocephalus* South + *P. kindae* v northern clade	1.99 (1.72–2.27)	1.76 (1.49–2.03)	
*P. ursinus* North + *P. cynocephalus* South − *P. kindae*	1.45 (1.19–1.72)	1.31 (1.06–1.56)	1.49 (1.03–1.98)
*P. ursinus* North − *P. cynocephalus* South	0.74 (0.55–0.94)	0.68 (0.51–0.87)	0.94 (0.58–1.30)
northwestern − northeastern clade	1.50 (1.26–1.74)	1.34 (1.11–1.57)	1.89 (1.33–2.48)
*P. anubis* West2 + *P. papio* − *P. anubis* West1	1.26 (1.03–1.48)	1.12 (0.91–1.35)	1.50 (1.02–2.02)
*P. anubis* West2 − *P. papio*	1.19 (0.97–1.41)	1.04 (0.82–1.26)	1.36 (0.91–1.86)
*P. hamadryas* + *P. anubis* East − *P. cynocephalus* North	0.39 (0.29–0.49)	0.31 (0.22–0.39)	
*P. hamadryas − P. anubis* East	0.23 (0.16–0.30)	0.16 (0.10–0.23)	

Estimates based on “Brown region” and cytochrome *b* sequence information are taken from Zinner et al. (2009).

## DISCUSSION

We aimed to find stronger statistical support in the phylogenetic tree reconstruction of *Papio* mitochondrial DNA, in particular for the most basal splits, and to narrow down the divergence time credibility intervals by basing our study on whole mitochondrial genomes. The second aim was accomplished by using complete mitochondrial genome information, whereas the resolution of the basal splits remained ambiguous.

The *Papio* phylogeny based on whole mitochondrial genome sequences shows a highly similar tree topology as the phylogeny based on sequences of the cytochrome *b* gene and the “Brown region” (Zinner et al.,^2009^, 2011a; Keller etal.,^2010^), but with stronger support for most nodes. Para- and polyphyletic relationships for almost all baboon species were confirmed. One possible cause for the presence of para- and polyphylies are “numts.” They can be excluded here, because overlapping parts of the various amplification fragments were identical, and because of the correct translation of protein-coding genes and the forming of typical secondary structures of tRNAs and rRNAs (Thalmann et al.,^2004^). Incomplete lineage sorting is also highly unlikely, because this process should be random in respect to geography (Avise,^2004^). However, in our mitochondrial phylogeny, we found that geographic close lineages cluster together, and, hence, introgressive hybridization remains as the most probable process leading to the observed phylogenetic discordances (Funk and Omland,^2003^; Burell^2008^; Zinner et al.,^2009^, 2011b; Jolly et al.,^2011^).

Estimated divergence times for all nodes are in a similar range as dates from other molecular studies (Chan et al.,^2010^; Perelman et al.,^2011^; Roos et al.,^2011^; Liedigk et al.,^2012^; Steiper and Seiffert,^2012^). Also among baboon lineages, estimated divergence times are generally consistent with previous work by Zinner et al. (2009), but appear slightly younger (Table 2). The respective credibility intervals have narrowed to <55%. For example, the breadth of the credibility interval around the northeastern and northwestern divergence, estimated at 1.50 Ma from dataset mtDNA1 and 1.34 Ma from dataset mtDNA2, is reduced to 95% CIs of only 0.48 and 0.46 Ma, respectively, and thus less wide than the 95% CI of 1.15 Ma around the previously estimated divergence time of 1.89 Ma (Zinner et al.,^2009^).

Although the tree topology in general is strongly supported by both algorithms, some relationships identified as weakly supported in earlier studies remained ambiguous (relationship among the three western clades, *P. anubis* West1, *P. anubis* West2, and *P. papio*, and the relationship between *P. ursinus* South and all other clades). The origin of the genus lies most likely in southern Africa. This is in agreement with earlier suggestions from mitochondrial phylogenies (Newman et al.,^2004^; Sithaldeen et al.,^2009^; Zinner et al.,^2009^; Keller etal.,^2010^) and fossil data (Jablonski and Frost,^2010^; Williams et al.,^2012^). However, the chronology of the initial divergences in southern Africa is not clear, because alternative scenarios are statistically not rejected. In our reconstruction, *P. ursinus* South diverged first followed by the main south–north split, but similarly possible are an initial south–north split or a trifurcation within a relative short time period (mtDNA1: 2.21–1.99 Ma; mtDNA2: 1.96–1.76 Ma) among the lineages leading to *P. ursinus* South, the clade consisting of *P. ursinus* North, *P. cynocephalus* South and *P. kindae*, and the northern clade.

The refined divergence dates among baboon mitochondrial lineages do not contradict the earlier hypothesis that the timing of divergence events among *Papio* lineages can be placed in a wider context related to changes in the African paleoclimate during the Pleistocene with recurrent expansions and retreats of the savanna biome as suitable habitat for baboons (Hamilton and Taylor,^1991^; deMenocal,^1995^, ^2004^; Maley,^1996^; Zinner et al.,^2009^, 2011a, Bettridge and Dunbar,^2012^). Because of periodical climate changes and the isolation and reconnection of savanna habitats, populations of baboons changed in size and spatial distribution, perhaps cyclically. Gene flow between populations occurred at differing degrees and at different times, leading to the phylogeographic pattern of baboons observed today (Zinner et al.^2009^, 2011a). Although key aspects of African faunal evolution in relation to climate change remain poorly understood (Bobé et al.,^2002^), previous studies have already pointed out the role of glacial and interglacial changes for refugial differentiation and migration routes for the African continent (Arctander et al.,^1999^; Nersting and Arctander,^2001^; Nyakaana et al.,^2002^; Lorenzen etal.,^2010^, ^2012^).

We are aware of the fact that with only mitochondrial DNA information at hand and the indications of excessive introgression, we cannot say much about the taxonomic level of the various baboon forms. However, a classification of baboon taxa as subspecies instead of species or as members of a superspecies would not change the general problem of finding closely related mitochondrial DNA in different taxa or largely different mitochondrial DNA in the same taxon. We would just shift the problem to another taxonomic level.

In conclusion, the general tree topology including paraphyletic relationships of most baboon species (Newman et al.,^2004^; Zinner et al.,^2009^; Keller et al.,^2010^) was confirmed by the use of whole mitochondrial sequence information and divergence times among baboon mitochondrial lineages became more reliable. This might have consequences for the use of *Papio* baboons as an analogous phylogeographic model for intra-African dispersal of hominins during the Pleistocene (Lahr and Foley,^1998^). However, to fully elucidate the putative complex evolutionary history of baboon taxa and to confirm hybridization events among them, large-scale nuclear sequence data are needed.
