# Supplementary material for: Baboon phylogeny as inferred from complete mitochondrial genomes
Source: Am J Phys Anthropol. 2013 Jan;150(1):133–40. doi: 10.1002/ajpa.22185 (PMC3572579; doi:10.1002/ajpa.22185)
Supplement: Supplementary file 1 [file ajpa0150-0133-sd1.docx]

Table S1: Information about length and selected substitution models of datasets and partitions.

| **Dataset** | **Partition** | **Length (bp)** | **Substitution model** |
| --- | --- | --- | --- |
| mtDNA1 | - | 16,055 | TIM2+I+G |
| mtDNA2 |  | 10,854 | - |
| mtDNA2 | ND1 | 954 | HKY+G |
| mtDNA2 | ND2 | 1,041 | TrN+I+G |
| mtDNA2 | COI | 1,536 | TrN+I+G |
| mtDNA2 | COII | 684 | HKY+G |
| mtDNA2 | ATP8 | 207 | HKY+I |
| mtDNA2 | ATP6 | 681 | TrN+G |
| mtDNA2 | COIII | 783 | HKY+I+G |
| mtDNA2 | ND3 | 345 | TrN+I+G |
| mtDNA2 | ND4L | 297 | TrN+I |
| mtDNA2 | ND4 | 1,377 | TrN+I+G |
| mtDNA2 | ND5 | 1,809 | TrN+I+G |
| mtDNA2 | Cytb | 1140 | HKY+G |
